# Early prediction of immunotherapy efficacy for advanced NSCLC based on clinical and pre-treatment contrast-enhanced CT radiomics features

**DOI:** 10.3389/fonc.2025.1711402

**Published:** 2025-12-19

**Authors:** Yue Hou, Tianming Zhang, Kaibo Zhu, Jing Jiang, Hong Wang

**Affiliations:** 1 Respiratory and Critical Care Medicine Department, Lanzhou University Second Hospital, Lanzhou, Gansu, China; 2 Second Clinical Medical College of Lanzhou University, Lanzhou, Gansu, China; 3 Radiology Department, Lanzhou University Second Hospital, Lanzhou, Gansu, China

**Keywords:** immunotherapy, machine learning, non-small cell lung cancer, radiomics, response prediction

## Abstract

**Background and purpose:**

To explore the predictive value of a model based on clinical and contrast-enhanced computed tomography (CT) radiomic features for the early prediction of immunotherapy efficacy in patients with advanced non-small cell lung cancer (NSCLC).

**Methods:**

This retrospective study included 144 patients with advanced NSCLC who received immunotherapy at Lanzhou University Second Hospital between January 2023 and December 2024. Clinical data and CT images were collected from each patient. All patients underwent imaging examinations to evaluate the efficacy of immunotherapy after the second treatment cycle. Patients who achieved complete response (CR) or partial response (PR) were considered to be in the reactive group, while those who experienced stable disease (SD) or progressive disease (PD) were considered to be in the non-reactive group. The participants were randomly divided into a training set (n = 115) and a testing set (n = 29) at a ratio of 8:2. Radiomic features were extracted from pre-treatment contrast-enhanced CT venous phase images. Feature reduction was performed using the Spearman rank correlation coefficient and the least absolute shrinkage and selection operator (LASSO) algorithm. The best radiomics signature was built using multiple machine learning algorithms and combined with clinical features to build a nomogram model. The area under the receiver operating characteristic curve (AUC), calibration curve, and decision curve analysis (DCA) were used to evaluate the model’s predictive performance, calibration, and clinical net benefit.

**Results:**

Three clinical features (C-reactive protein, baseline tumor size, and programmed death receptor ligand 1) and seven radiomics features (one first-order feature and six texture features) were selected for the model. The radiomic signature performed best based on the Extreme Random Tree algorithm. The radiomic signature and the nomogram model demonstrated superior predictive performance and clinical net benefit compared to the clinical model in both training and testing sets (AUCs: radiomics: 0.926 vs. 0.848; nomogram: 0.953 vs. 0.788; clinical: 0.882 vs. 0.742), with statistically significant differences (P < 0.05).

**Conclusion:**

The integrated clinical-radiomics nomogram establishes a robust framework for early prediction of immunotherapy efficacy in advanced NSCLC, offering valuable support for personalized treatment decisions.

## 1 Introduction

Non-small cell lung cancer (NSCLC) is the leading cause of cancer-related death worldwide. The five-year survival rate for patients with advanced NSCLC is less than 5% owing to the loss of surgical opportunities, limited efficacy of radiotherapy and chemotherapy, and significant toxicity (1, 2). In recent years, immunotherapy has emerged as a first-line treatment for advanced NSCLC patients without driver mutations, significantly improving the five-year survival rate to 23.5% (3, 4). However, the efficacy of currently clinically applied predictive biomarkers, such as programmed cell death ligand 1 (PD-L1) and tumor mutational burden (TMB), is limited by sampling limitations and tumor spatial heterogeneity. Furthermore, PD-L1 expression and the efficacy of immunotherapy are influenced by treatment regimens, tumor heterogeneity, and tumor microenvironment, resulting in only a few patients benefiting from it in the long term (5). Therefore, it is necessary to accurately identify individuals who are sensitive to immune treatment. This is important for guiding decisions regarding late-stage NSCLC treatment. The goal is to extend the patients’ lives.

Radiomics is a process that uses computer software to extract high-throughput features from traditional imaging data. This method avoids the limitations of invasive tissue biopsy, such as missing tumor spatial heterogeneity and poor reproducibility. It can comprehensively reflect tumor biology and provide safer and more reliable guidance for patient follow-up and prognosis monitoring in the future. In 2018, Sun Roger (6, 7) first demonstrated that radiomics could be used to predict the efficacy of immunotherapy. Since then, radiomics research has focused on predicting the prognosis, evaluating the efficacy, and monitoring the immunotherapy-related adverse reactions of different tumors. This study aimed to explore the predictive value of clinical characteristics and treatment-before-enhanced CT radiomics features for the early prediction of the efficacy of immunotherapy in late-stage NSCLC. The goal is to establish a predictive model related to efficacy and provide an early, non-invasive, and high-precision predictive tool for individualized immunotherapy decisions.

## 2 Materials and methods

### 2.1 Patients

**Figure 1:**
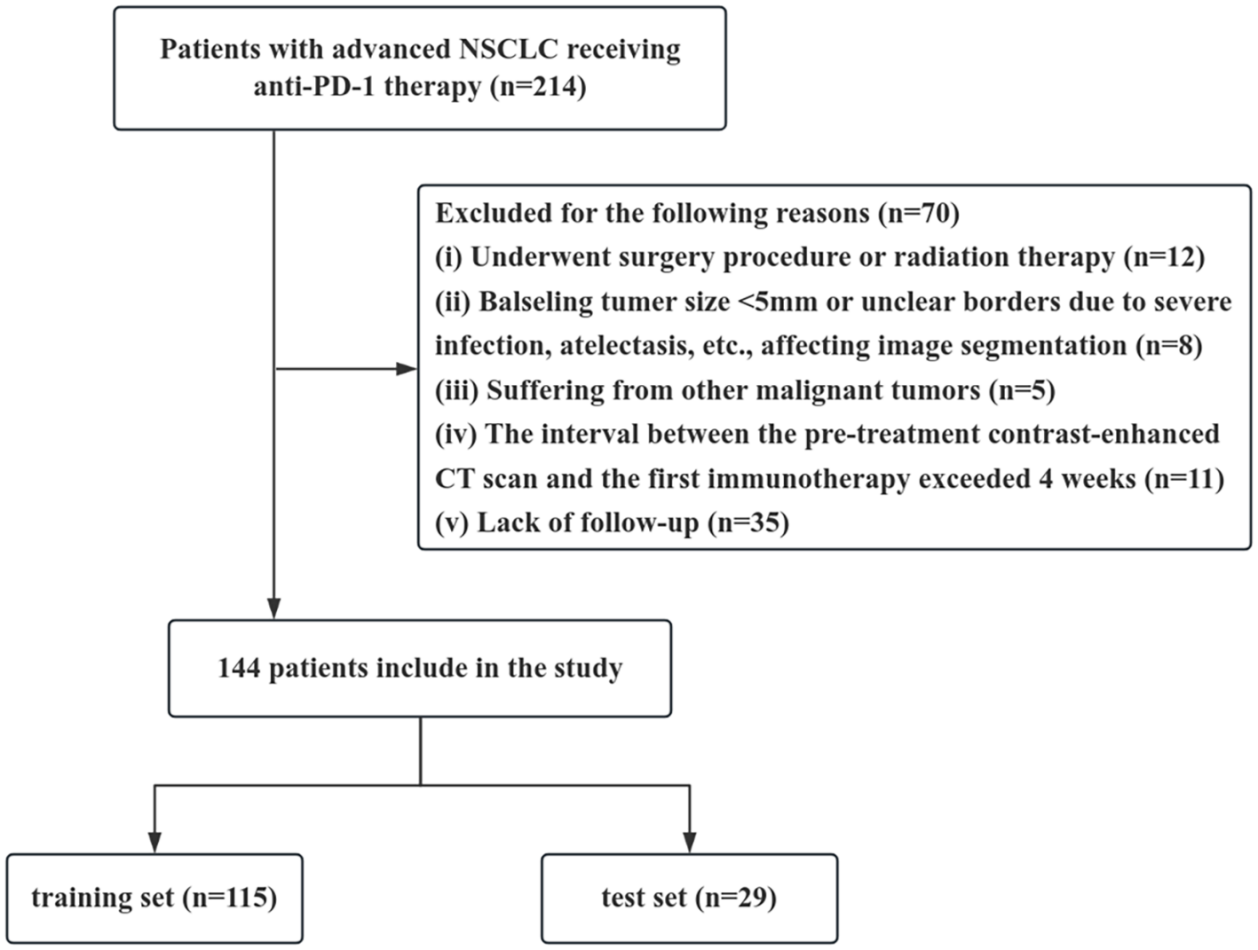
Patient flow diagram. For the study dataset, training and test set were randomly divided in a proportion of 8:2. NSCLC, Non-small cell lung cancer; CT, computed tomography.

This retrospective study included 144 patients with advanced non-small cell lung cancer (NSCLC) who received immune checkpoint inhibitor (ICI) treatment at Lanzhou University Second Hospital between January 2023 and December 2024. The inclusion criteria were as follows: (1) TNM stage IIIb to IV; (2) pathologically confirmed NSCLC; (3) at least two cycles of first-line PD-1 inhibitor combined with chemotherapy; and (4) PS score 0-2. The exclusion criteria were as follows: (1) receiving radiotherapy or surgery before immunotherapy; (2) maximum tumor diameter <5 mm or unclear tumor border due to lung infection, lung collapse, etc., affecting image segmentation; (3) other malignant tumors; (4) interval between pre-treatment enhanced CT and immunotherapy was greater than 4 weeks; and (5) incomplete data or lost follow-up. The enrollment process is illustrated (**Figure 1**). The study was approved by the Ethics Committee (2024A-306), and informed consent was waived.

### 2.2 Chest enhanced CT

Imaging Protocol The scanning equipment utilized in this study included the Siemens Somatom Force CT, Philips Brilliance iCT, GE Discovery HD750, and GE Revulation CT. The scanning parameters are listed in **Table 1**. The scanning method was as follows: the patient assumed a supine position with both arms raised above the head. Following deep inhalation, the patient was asked to hold their breath for a period of time. The scanning range encompassed the cranium and inferior border of the ilium. Subsequently, the patient was administered 1.5 mL of a contrast agent containing 320 mg of iodine per milliliter (IsoTec, Bayer AG) via a high-pressure injector. The patient was asked to collect an arterial phase image 30 s after the injection of the contrast agent and a venous phase image at 60 s.

**Table 1:** Scanning parameters for scanner devices.

Devices	Tube voltage	Tube current	Collimation	Rotational speed	Rotation speed	Layer thickness	Interlayer gap
Siemens Somatom Force CT	120KV	Automatic mAs technology	0.6 mm	0.5 s/rot	1.2	1 mm	1 mm
Philips Brilliance iCT	120KV	Automatic mAs technology	0.625 mm	0.5 s/rot	1.15	1.5 mm	1 mm
GE Discovery HD750	80/140kVp	275mA	0.625 mm	0.7 s/rot	1.375	1.25 mm	1.25 mm
GE Revolution CT	80/140kV	400mA	0.625 mm	0.8 s/rot	0.992	1.25 mm	1.25 mm

### 2.3 Data collection and efficacy evaluation of immunotherapy

The clinical dataset included all patients who underwent combination chemotherapy with platinum derivatives and immune checkpoint inhibitors (ICIs) as a treatment modality. The immunotherapy regimen consisted of a PD-1 inhibitor. The patients’ baseline characteristics were collected using the hospital’s electronic medical records system. These characteristics included age, race, sex, height, weight, body mass index (BMI), smoking history, family history of cancer, TNM staging, pathological type, and Eastern Cooperative Oncology Group performance status (ECOG PS) score. Baseline tumor size (BTS) was also recorded. Serum markers, including C-reactive protein (CRP), albumin, and Lactate Dehydrogenase (LDH), were also measured. Serum levels of Carcinoembryonic Antigen (CEA), Neuron-Specific Enolase (NSE), cytokeratin 19 Fragment Antigen 21-1 (CYFRA21-1), progastrin-releasing peptide (ProGRP), and Squamous Cell Carcinoma Antigen (SCC) were also measured. PD-L1 expression was detected using immunohistochemistry (IHC) on lung tissue samples obtained via bronchoscopy or CT-guided needle biopsies. The evaluation of at least 100 tumor cells (TCs) is necessary, and the tumor cell percentage (TPS) should be used for quantitative analysis (8). TPS was categorized as follows: negative (TPS < 1%), low (TPS 1%-49%), and high (TPS ≥ 50%).

The following images were obtained using a picture archiving and communication system (PACS) in the Digital Imaging and Communications in Medicine (DICOM) format. The images were obtained during the chemotherapy and immunotherapy treatment periods, specifically during the first four weeks of intravenous infusion. The images were analyzed using image analysis software.

**Figure 2:**
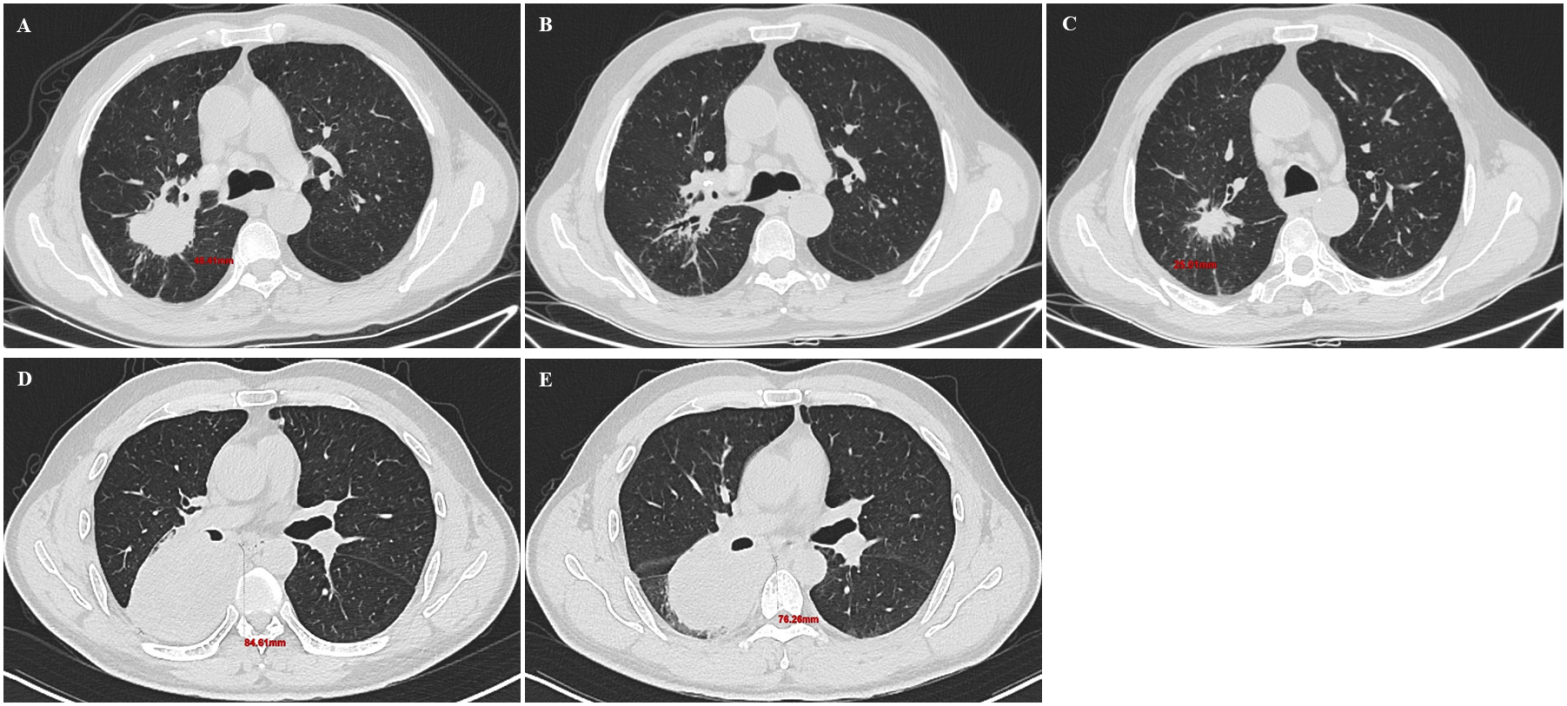
Imaging findings at the first on-treatment evaluation demonstrating Partial Response (PR) and Stable Disease (SD). PR: A 68-year-old male with right lung SCC. Baseline scan shows a target lesion (48.41 mm) **(A)**. At first follow-up, the lesion is not measurable at the same level **(B)**, the residual lesion measures 25.01 mm **(C)**. SD: A 48-year-old male with right lung SCC. Baseline scan shows a target lesion (84.61 mm) **(D)**. At first follow-up, the lesion measures 76.26 mm **(E)**.

**Figure 3:**
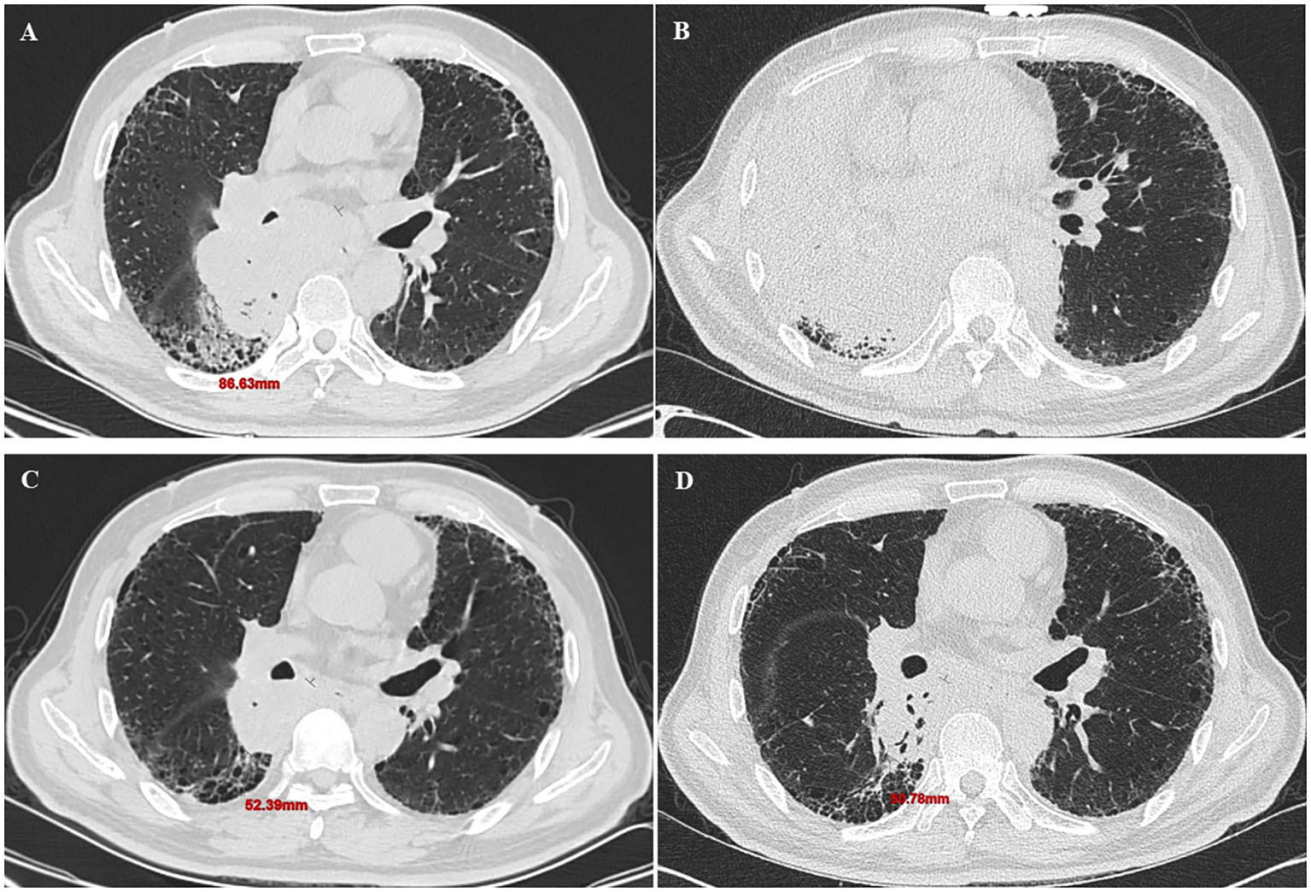
Individualized management and serial imaging for a patient initially assessed with iUPD. A 63-year-old man with right lung adenocarcinoma. Baseline scan demonstrates the target lesion (86.63 mm) **(A)**. After 2 cycles of chemoimmunotherapy, the first follow-up showed iUPD **(B)**. Despite progression, treatment was continued due to suspected pseudoprogression. After a third cycle, a subsequent scan revealed significant tumor shrinkage to 52.39 mm, confirming the iUPD was unconfirmed **(C)**. After 6 cycles, tumor size was 50.78 mm, confirming a best overall response of iPR **(D)**.

Efficacy evaluation of immunotherapy: The primary endpoint of this study was the final immunotherapy response, determined based on the initial radiological assessment followed by necessary confirmatory procedures. All patients underwent baseline contrast-enhanced chest CT and routine laboratory tests within 4 weeks before initiating immunotherapy. The first follow-up imaging evaluation was performed at 6–8 weeks after the initial treatment. Tumor response was strictly evaluated according to the iRECIST criteria (9). Complete response (iCR) was defined as the disappearance of all target lesions. Partial response (iPR) was defined as a ≥30% decrease in the sum of diameters of all target lesions relative to baseline. Stable disease (iSD) was defined as a change in the sum of diameters ranging from −30% to +20% (**Figure 2**). Unconfirmed progressive disease (iUPD) was defined as a ≥20% increase in the sum of diameters. For patients assessed as iUPD, a comprehensive evaluation was conducted by a respiratory physician to decide whether to continue the original treatment regimen. If treatment was continued, a confirmatory imaging scan was performed 4–6 weeks later. The final outcome (iCPD, iSD, or iPR) determined from this confirmatory scan was recorded as the study endpoint for that patient (**Figure 3**). For analysis purposes, patients were categorized into two groups: responders (best overall response of iCR or iPR, including those converting from iUPD to iPR) and non-responders (best overall response of iSD or iCPD).

### 2.4 Image analysis

#### 2.4.1 Image preprocessing and segmentation

The images were resampled to a uniform voxel size of 1×1×1 mm³ using linear interpolation. Gray levels were discretized via min-max normalization into 25 fixed intensity bins (10). The preprocessed images were then segmented using the ITK-SNAP software (http://www.itksnap.org). Initially, a junior physician (Yue HOU) manually delineated the region of interest (ROI) on the transverse plane displaying the maximum tumor diameter, using a lung window setting (width: 1500 HU; level: -500 HU). All these initial ROIs were subsequently reviewed and revised by a senior respiratory specialist (Tianming ZHANG). The delineation was guided by the tumor-lung interface, carefully excluding necrotic or calcified areas, adjacent vessels, and peripheral non-tumor regions (**Supplementary Figure 1**). To assess the inter- and intra-observer consistency of the segmentation method, two senior experts (Tianming ZHANG and Kaibo ZHU) independently delineated the lesions in a randomly selected cohort of 30 images following the same protocol.

#### 2.4.2 Radiomics feature extraction, screening, and modeling

**Figure 4:**
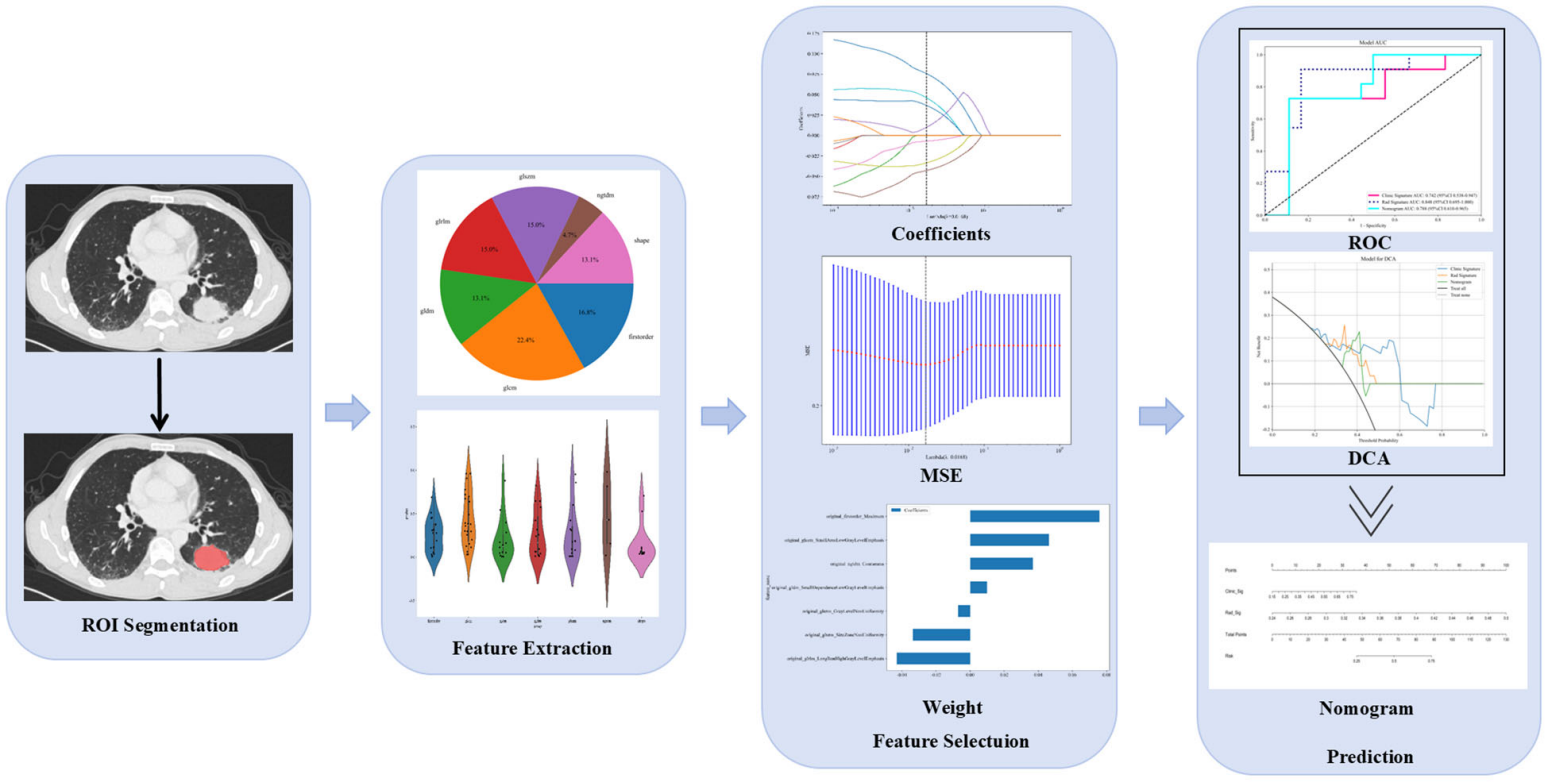
Workflow for the radiomics analysis. ROI, region of interest; MSE, mean squared error; ROC, receiver operator characteristic; DCA, decision curve analysis.

Imaging features were extracted from the ROIs using the Python PyRadiomics package (http://pyradiomics.readthedocs.io/). Z-score normalization was first applied to standardize the data. Inter- and intra-observer consistency of features extracted by the two experts were then evaluated, and only radiomic features demonstrating high reproducibility (ICC ≥ 0.75) were retained. A Mann–Whitney U test was performed on all features, and those with a p-value below 0.05 were kept. Subsequently, to minimize redundancy, features exhibiting a Spearman’s correlation coefficient greater than 0.9 were excluded. The dataset was randomly divided into a training set (115 cases) and a testing set (29 cases), approximating an 8:2 ratio. On the training set, feature dimensionality was reduced using the least absolute shrinkage and selection operator (LASSO). The optimal hyperparameter (α) for LASSO was determined via five-fold cross-validation applied exclusively to the training set. The final feature set, consisting of features with nonzero coefficients, was obtained by fitting a LASSO model on the entire training set using this optimal α. These selected imaging features were used to construct a Rad-score via multiple machine learning algorithms, including logistic regression (LR), support vector machines (SVM), random forest (RF), and extreme randomized trees (ET). Similarly, significant clinical features (P < 0.05) were incorporated into a clinical model. The best-performing algorithm was selected to integrate the Rad-score and clinical features into a combined prediction model (8, 11–13), which was ultimately presented as a nomogram for clinical application. The overall workflow is depicted in **Figure 4**.

### 2.5 Internal validation of the nomogram

To provide a robust and unbiased estimate of the nomogram’s generalizability and to address potential overfitting, the model was internally validated using a repeated stratified 5-fold cross-validation scheme on the training set (n=115). With 50 repetitions, this process yielded 250 performance estimates. These estimates were used to comprehensively assess the model’s stability and predictive performance.

### 2.6 Statistical analysis

Statistical analyses were performed using R software version 4.3.3. Normally distributed quantitative data were expressed as mean ± standard deviation (*X ± s)*, and intergroup comparisons were conducted using the independent samples t-test. Non-normally distributed quantitative data were expressed as median (interquartile range) [M(P25,P75)], and intergroup comparisons were performed using the Mann-Whitney U test. Categorical data were expressed as percentages (%). Intergroup comparisons were performed using chi-square (*χ^2^*) and Fisher’s exact tests. Differences were considered statistically significant at P < 0.05. Receiver operating characteristic (ROC) curves were plotted, and the area under the curve (AUC) was calculated to assess the predictive performance of the models. The sensitivity, specificity, and accuracy were also computed. The DeLong test was used to compare the performance differences among the different models. Calibration curves were used to assess the consistency between the predicted probabilities and actual outcomes. The Hosmer-Lemeshow test was used to evaluate the goodness-of-fit of the models. Decision curve analysis (DCA) was conducted to assess the clinical utility of the models (11–14). Furthermore, to enable a direct quantitative comparison at specific clinical decision points, we calculated the net benefit, sensitivity, specificity, positive predictive value (PPV), and negative predictive value (NPV) for each model at the key thresholds of 0.2, 0.3, and 0.5.

## 3 Results

### 3.1 Comparing the clinical characteristics of patients

According to the inclusion and exclusion criteria, this study included 144 patients, aged 36–86 years, with a median age of 61.00 years. Among them, 88 patients (61.11%) were 60 years or older, and 56 patients (38.89%) were younger than 60 years. There were 132 males and 12 females, with 97 patients having squamous cell carcinoma and 47 patients having adenocarcinomas. The patients were randomly divided into a training set of 115 patients (72 with a response and 43 without a response) and a testing set of 29 patients (18 with a response and 11 without a response). The results showed statistically significant differences in CRP, PD-L1, and BTS in the training set (P<0.05). The other demographic characteristics (age, gender, race, etc.), smoking history, family history of cancer, and TNM stage did not differ and were not statistically significant (P > 0.05), as shown in **Table 2**.

**Table 2:** Characteristics of patients included in the study and P values revealing statistical differences between the study cohorts.

Variables		Total set (n = 144)	Training set		*P*	Testing set		*P*
			Reactive group (n = 72)	Non-reactive group (n = 43)		Reactive group (n = 18)	Non-reactive group (n = 11)	
Age(years)		61.00 (56.75, 68.00)	61.00 (57.00, 68.00)	62.00 (58.00, 69.00)	0.466	63.50 (54.00, 65.75)	57.00 (56.00, 63.00)	0.685
BMI(kg/m^2^)		22.26 (20.42, 24.68)	22.19 (20.27, 24.39)	21.83 (20.25, 24.27)	0.954	23.10 (20.89, 25.34)	21.46 (20.48, 25.02)	0.418
Race	Han ethnic	130 (90.28)	65 (90.28)	39 (90.70)	0.058	17 (94.44)	9 (81.82)	0.135
	Other	14(9.72%)	7(9.72)	4 (9.30)		1 (5.56)	2 (18.18)	
Sex	Male	132 (91.67)	68 (94.44)	36 (83.72)	0.118	17 (94.44)	11 (100.00)	1.000
	Female	12 (8.33)	4 (5.56)	7 (16.28)		1 (5.56)	0 (0.00)	
Smoking status	Never	67 (46.53)	33 (45.83)	21 (48.84)	0.755	9 (50.00)	4 (36.36)	0.702
	Former	77 (53.47)	39 (54.17)	22 (51.16)		9 (50.00)	7 (63.64)	
Family history of cancer	No	139 (96.53)	70 (97.22)	42 (97.67)	1.000	18 (100.00)	9 (81.82)	0.135
	Yes	5 (3.47)	2 (2.78)	1 (2.33)		0 (0.00)	2 (18.18)	
cT	T1	8(5.56)	5 (6.94)	2 (4.65)	0.833	1 (5.56)	0 (0.00)	0.658
	T2	35(24.31)	17 (23.61)	11 (25.58)		3 (16.67)	4 (36.36)	
	T3	28(19.44)	14 (19.44)	9 (20.93)		4 (22.22)	1 (9.09)	
	T4	73(50.69)	36 (50.00)	21 (48.84)		10 (55.56)	6 (54.55)	
cN	N0	21(14.58)	9 (12.50)	7 (16.28)	0.085	2 (11.11)	3 (27.27)	0.642
	N1	19(13.19)	11 (15.28)	2 (4.65)		4 (22.22)	2 (18.18)	
	N2	60(41.67)	32 (44.44)	18 (41.86)		7 (38.89)	3 (27.27)	
	N3	44(30.56)	20 (27.78)	16 (37.21)		5 (27.78)	3 (27.27)	
cM	M0	80(55.56)	41 (56.94)	25 (58.14)	0.990	8 (44.44)	6 (54.55)	0.672
	M1a	20(13.89)	11 (15.28)	5 (11.63)		2 (11.11)	2 (18.18)	
	M1b	15(10.42)	6 (8.33)	3 (6.98)		5 (27.78)	1 (9.09)	
	M1c	29(20.14)	14 (19.44)	10 (23.26)		3 (16.67)	2 (18.18)	
Histology	SCC	97 (67.36)	45 (62.50)	31 (72.09)	0.293	13 (72.22)	8 (72.73)	1.000
	ADC	47 (32.64)	27 (37.50)	12 (27.91)		5 (27.78)	3 (27.27)	
ECOG PS score	0	10(6.94)	5 (6.94)	1 (2.33)	0.620	1 (5.56)	3 (27.27)	0.198
	1	98(68.06)	50 (69.44)	31 (72.09)		12 (66.67)	5 (45.45)	
	2	36(25.00)	17 (23.61)	11 (25.58)		5 (27.78)	3 (27.27)	
TPS(%)	<1	49(34.03	13 (18.06)	24 (55.81)	**<.001**	5 (27.78)	7 (63.64)	0.328
	1-49	30(20.83)	15 (20.83)	9 (20.93)		5 (27.78)	1 (9.09)	
	≥50	65(45.14)	44 (61.11)	10 (23.26)		8 (44.44)	3 (27.27)	
Ki67(%)		50.00 (40.00, 70.00)	60.00 (42.50, 70.00)	60.00 (40.00, 70.00)	0.814	42.50 (32.50, 70.00)	50.00 (45.00, 60.00)	0.206
CRP(mg/L)		12.62 (2.56, 43.15)	25.81 ± 24.23	47.73 ± 61.00	**0.033**	40.95 ± 50.88	36.93 ± 42.64	0.856
Albumin(g/L)		39.50 (36.70, 41.65)	39.40 (36.77, 41.45)	39.50 (35.47, 41.68)	0.974	39.20 (37.40, 41.17)	39.80 (36.90, 44.45)	0.574
LDH(U/L)		215.00 (186.00, 251.50)	211.00 (186.50, 251.50)	213.00 (187.50, 262.50)	0.601	215.50 (160.00, 254.50)	224.00 (214.00, 233.00)	0.571
CEA(ng/mL)		2.87 (1.69, 4.62)	3.54 (2.23, 6.43)	2.57 (1.70, 4.05)	0.269	3.27 (1.43, 5.20)	2.95 (1.20, 5.97)	0.633
NSE(ng/mL)		15.20 (12.85, 19.00)	18.15 (14.88, 22.75)	16.60 (13.60, 20.30)	0.309	19.45 (12.93, 22.23)	19.20 (14.10, 21.00)	1.000
CYFRA21-1(ng/mL)		2.50 (1.82, 3.65)	4.04 (2.64, 10.53)	3.44 (2.20, 7.67)	0.094	3.55 (2.54, 12.85)	4.71 (2.71, 14.80)	0.604
ProGRP(pg/mL)		44.80 (34.70, 58.30)	44.10 (33.12, 53.80)	47.20 (34.10, 57.30)	0.378	48.10 (40.10, 56.60)	35.05 (33.50, 41.53)	**0.046**
SCC(ng/mL)		1.25 (1.01, 1.98)	1.42 (0.99, 2.49)	1.47 (0.92, 4.01)	0.267	1.16 (1.03, 1.47)	1.32 (1.12, 2.40)	0.280
BTS(mm)		47.00 (36.00, 77.00)	44.50 (36.00, 58.25)	72.00 (37.00, 79.00)	**0.018**	45.00 (42.00, 74.00)	50.50 (42.50, 58.00)	1.000

**Table 2**, bolded P-values indicate statistical significance (P < 0.05).

### 3.2 Feature extraction and radiomics signature construction

**Figure 5:**
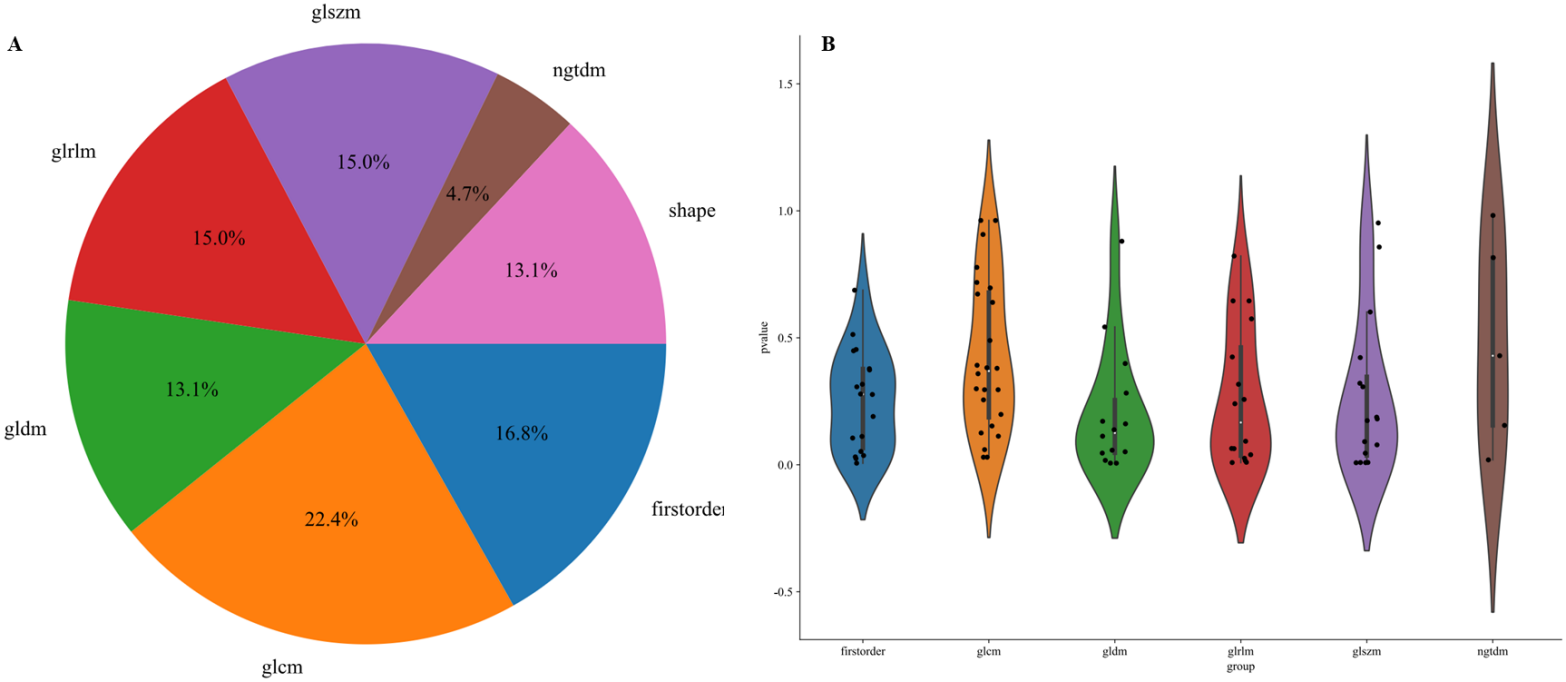
Ratio of handcrafted features **(A)**; Statistics of radiomic features **(B)**.

**Figure 6:**
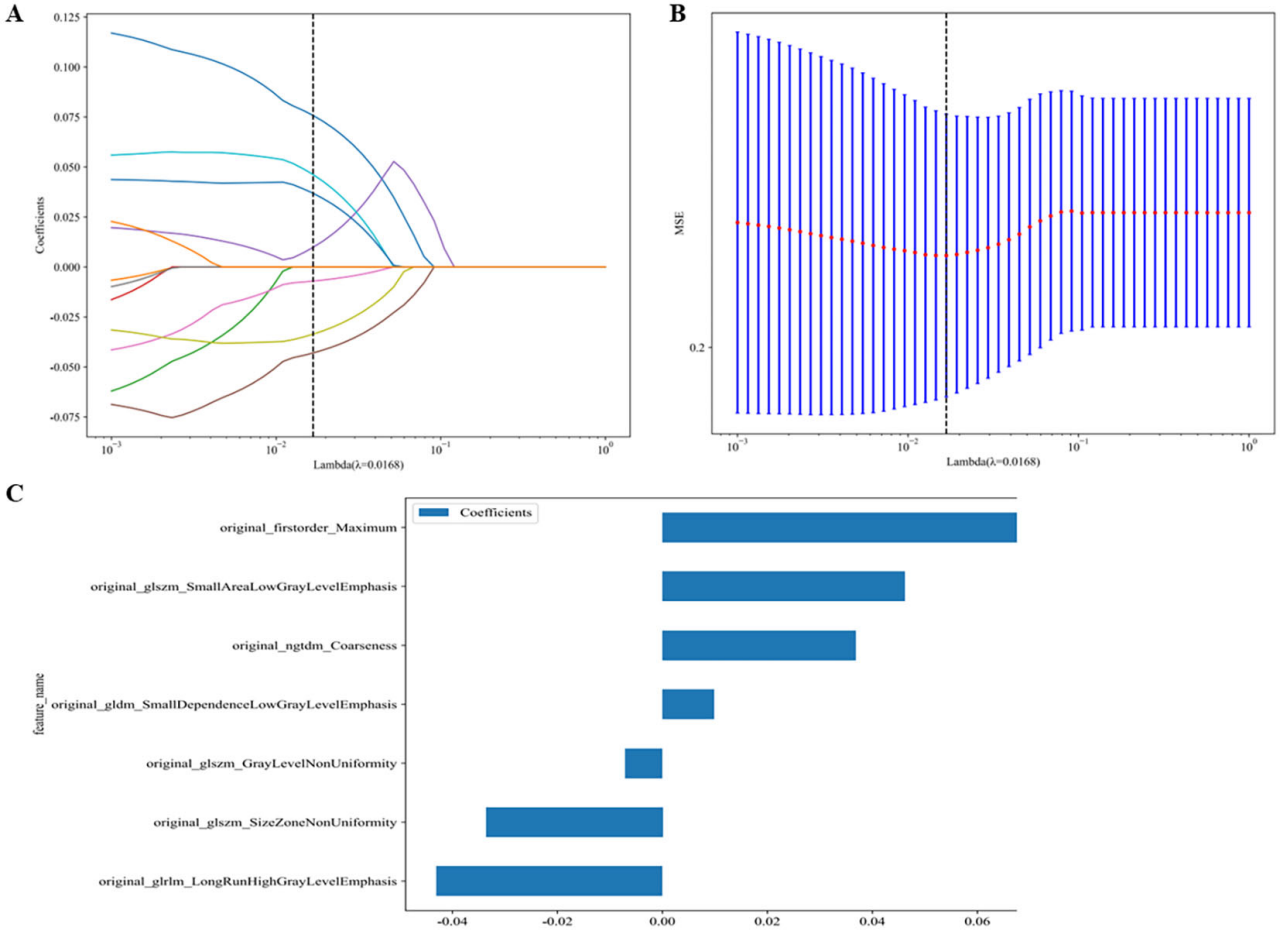
Coefficients of 5 fold cross validation **(A)**. MSE of 10 fold cross validation **(B)**. The histogram of the Rad-score based on the selected features **(C)**.

**Figure 7:**
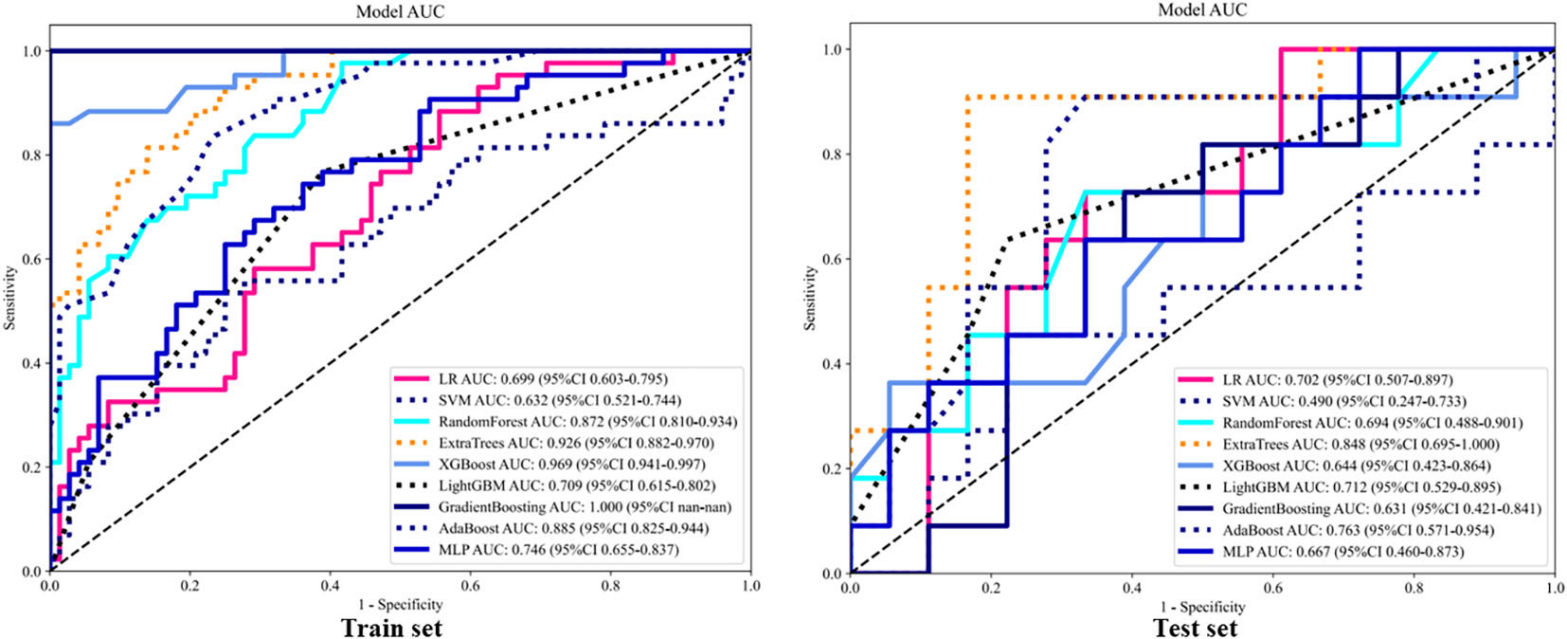
ROC analysis of different models on rad signature.

A total of 107 radiomics features were extracted, including 18 first-order features, 14 shape features, 5 NGTDM features, 16 GLSZM features, 16 GLRLM features, 14 GLDM features, and 24 GLCM features. Following univariate screening with the Mann-Whitney U test (p < 0.05), 24 significant features were identified. The percentage of each feature and its statistical values are presented in **Figure 5** Subsequent removal of highly correlated features (Spearman’s |ρ| > 0.9) reduced the number to 12. A LASSO regression model applied to these 12 features selected the final 7 most predictive features, whose coefficients and cross-validated MSE are presented. The features and their coefficients are shown in **Figure 6**. The optimal image set features were then used to construct a prediction model using machine learning methods, such as LR, SVM, random forest, and extra trees. **Supplementary Table 1** summarizes all models evaluated for predicting immunotherapy efficacy. Among them, the Extra Trees algorithm demonstrated superior performance, achieving the highest AUC values in both the training set (0.926; 95% CI: 0.882–0.970) and the testing set (0.848; 95% CI: 0.695–1.000), as illustrated in **Figure 7**. Consequently, it was selected as the final clinical prediction model. A radiomics score (Rad-score) was constructed using the LASSO logistic regression model with the following formula:Radscore= 0.375 + 0.075783 * original_firstorder_Maximum +0.009822 * original_gldm_SmallDependenceLowGrayLevelEmphasis -0.042976 * original_glrlm_LongRunHighGrayLevelEmphasis -0.007042 * original_glszm_GrayLevelNonUniformity -0.033593 * original_glszm_SizeZoneNonUniformity +0.046212 * original_glszm_SmallAreaLowGrayLevelEmphasis +0.036860 * original_ngtdm_Coarseness. P(Non-reactive=1) = 1/(1 + e^(-Z)), where Z = -2.80967098 + 3.16488672 × Clinic_Sig + 2.82782853 × Rad_Sig.

### 3.3 Comparing clinical, radiomic signature, and nomogram model

**Figure 8:**
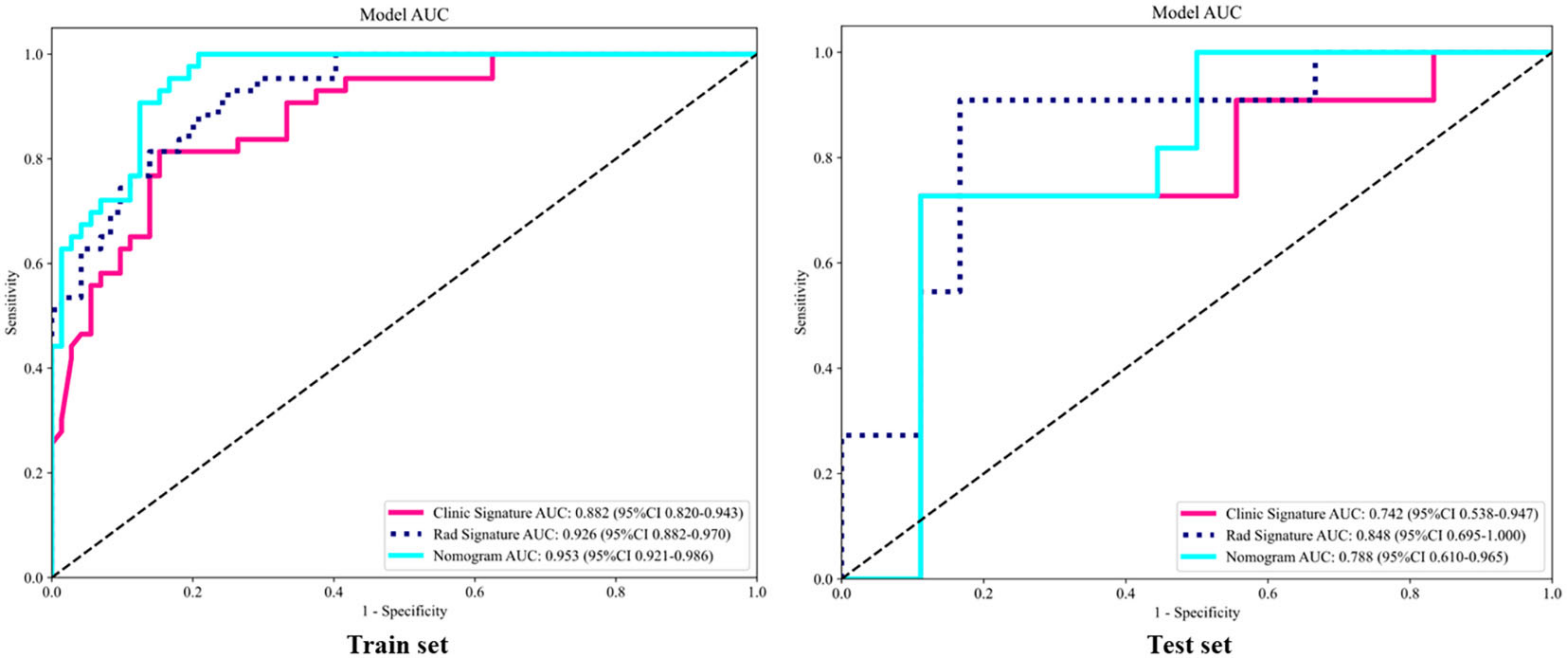
AUC comparison of clinical, radiomics and combined models. Compared with clinical and radiomics models, the combined model had the best performance in the training set and validation set.

**Figure 9:**
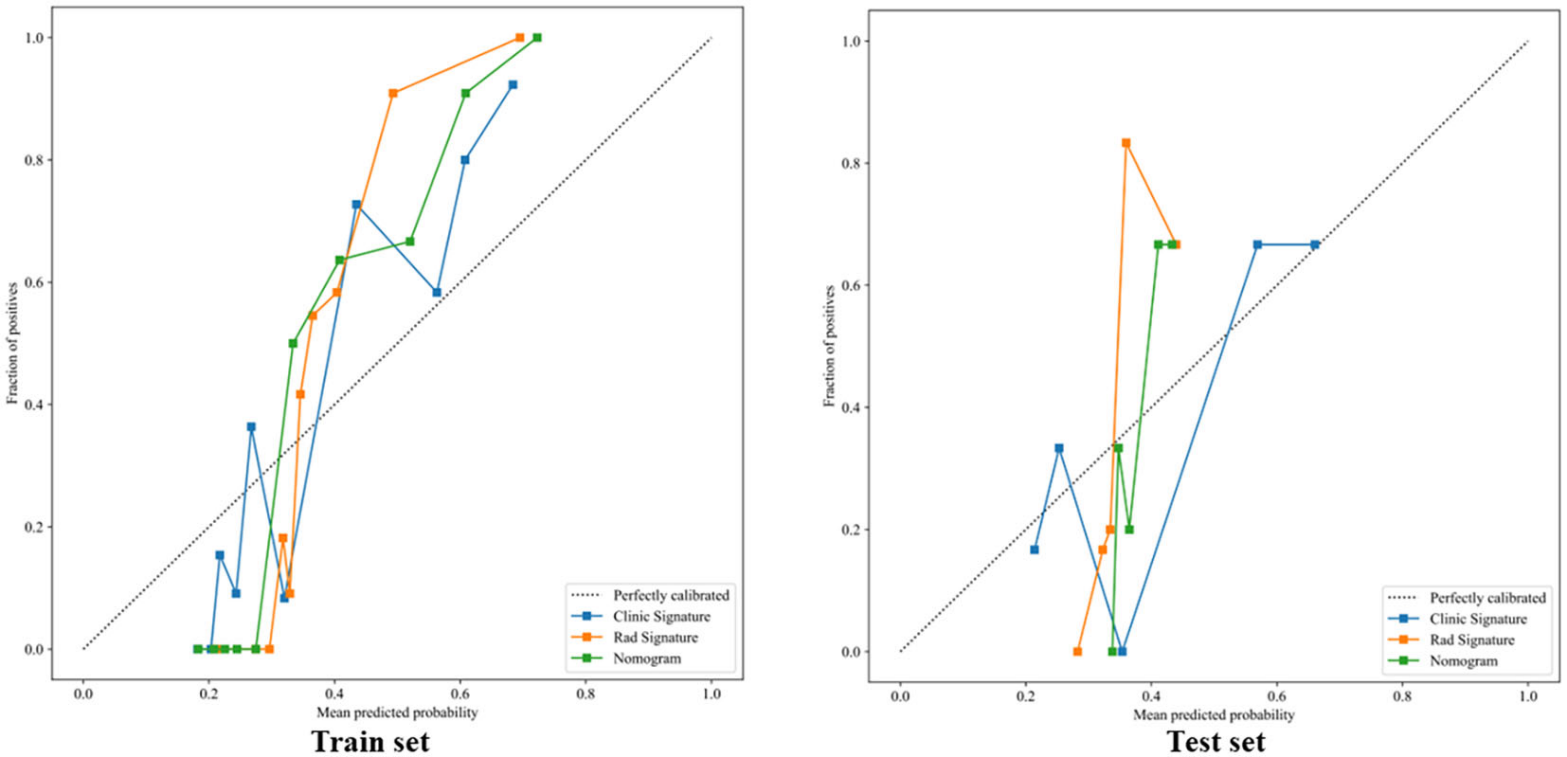
The calibration curves in train and test set.

The clinical, radiomics, and nomogram models all demonstrated good specificity in the training set, with the nomogram model achieving the highest predictive performance, the AUCs of 0.882 (95% CI: 0.820–0.943), 0.926 (95% CI: 0.882–0.970), and 0.953 (95% CI: 0.921–0.986), respectively. In the validation set, both the clinical and radiomics models showed good fit, whereas the nomogram model may be overfitted, with AUCs of 0.742 (95% CI: 0.538–0.947), 0.848 (95% CI: 0.695–1.000), and 0.788 (95% CI: 0.610–0.965). Although the radiomics model outperformed the nomogram model, the difference was not statistically significant (P > 0.05), as shown in **Figure 8**. Calibration assessment indicated room for improvement in the absolute accuracy of model predictions. Although the Hosmer–Lemeshow test P-values for the clinical model, radiomics signature, and nomogram in the validation set were all greater than 0.05 (0.354, 0.515, and 0.086, respectively), the calibration curves (**Figure 9**) revealed visible deviations between predicted and observed outcomes. Moreover, the nomogram’s P-value (0.086) approached the borderline of statistical significance. These findings suggest that the calibration performance of the models is suboptimal, and they may be more suitable for risk stratification than for precise probability estimation. Thus, the predicted probabilities should be interpreted as indicative of a relative risk range rather than as exact point estimates.

**Figure 10:**
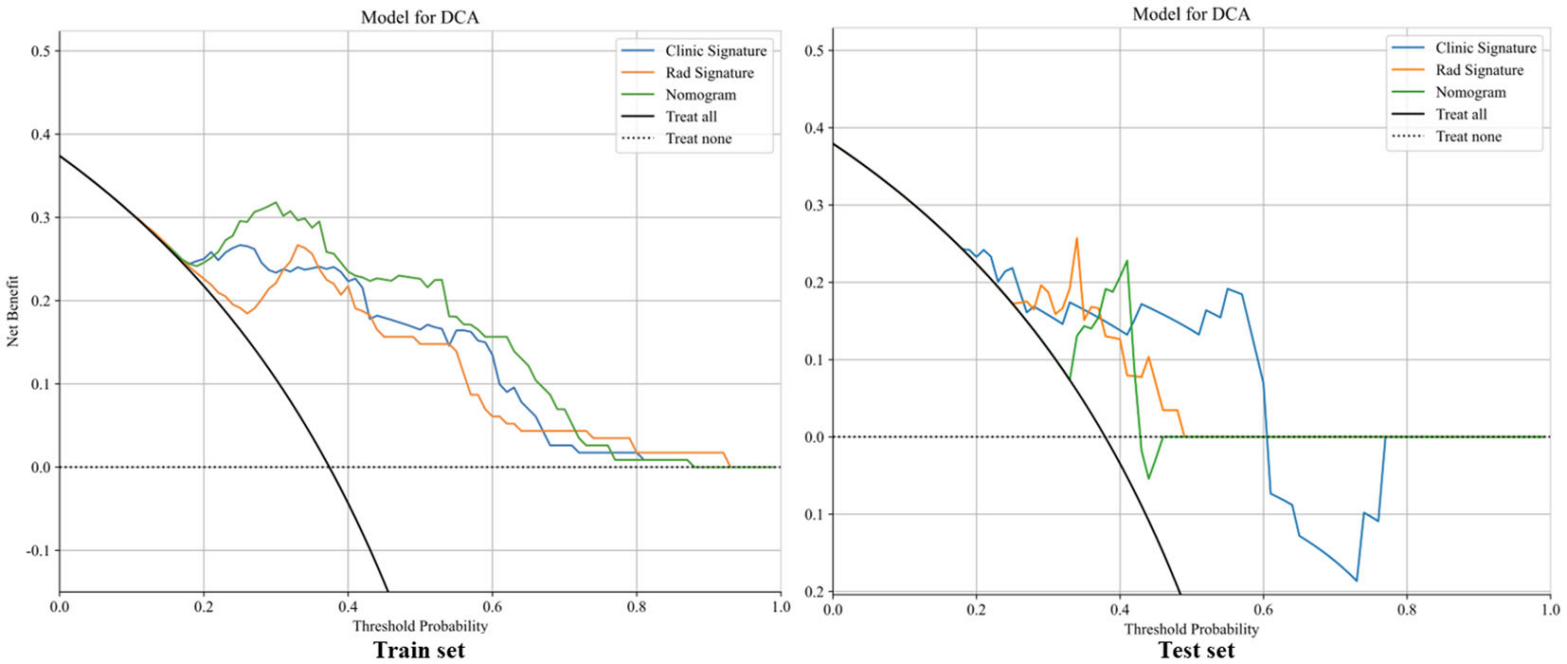
Decision curve of in in train and test test.

**Figure 11:**
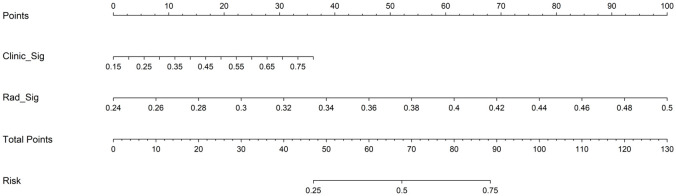
The CT-based radiomics nomogram for clinical use.

The decision curves for the clinical, radiomics, and nomogram models are presented in **Figure 10**. Quantitative clinical utility analysis demonstrated that at the probability threshold of 0.3, the nomogram achieved a net benefit of 0.318 with perfect sensitivity (100%) and specificity of 79.2%, significantly outperforming simple clinical baseline models including PD-L1 high expression (net benefit: 0.020), BTS <50 mm (net benefit: -0.063, indicating net harm), and low CRP (net benefit: 0.024), as detailed in **Supplementary Figure 4** and **Table 2**. The clinical application of the nomogram model is illustrated in **Figure 11**, where the total score reflects the probability of achieving PD or SD as the initial treatment response, providing quantifiable advantages for clinical decision-making. The comprehensive performance metrics for all final models are detailed in **Supplementary Table 2**.

### 3.4 Unbiased performance estimate by internal validation

The notable performance drop of the nomogram from the training set (AUC = 0.953) to the initial testing set (AUC = 0.788) suggested potential overfitting. To robustly estimate the model’s generalizable performance and address this concern, we performed a rigorous internal validation using 50 repeats of stratified 5-fold cross-validation on the training cohort (n=115).

This validation yielded a mean AUC of 0.783 (95% CI: 0.777 – 0.790), which aligns closely with the initial test set performance (AUC = 0.788). The high consistency between these independent assessments strongly indicates that the model’s generalizable discrimination ability is approximately 0.78, not the overly optimistic 0.953 observed on the training set. Furthermore, the low coefficient of variation (3.02%) and the detailed distribution of performance across all repetitions (**Supplementary Figure 2**) confirm the satisfactory stability of the model.

## 4 Discussion

In China, the incidence of lung cancer is increasing, and the burden of cancer is expected to continue to increase over the next 20 years. Despite the increasing number of treatment options, the prognosis for patients with lung cancer remains poor (2, 15). The objective response rate to immunotherapy varies among patients with NSCLC (16). Identifying patients who will benefit from immunotherapy early on and switching patients who do not respond to a different treatment plan early on is crucial for reducing the disease burden on patients. This study developed and validated a model based on clinical and pre-treatment enhanced CT imaging features that outperformed traditional clinical prediction models. This model can provide an early, noninvasive predictive tool for late-stage NSCLC immunotherapy decisions.

Immunotherapy outcomes are influenced by a multifactorial interplay of tumor-related factors (e.g., histology, metastatic pattern, BTS), host-related factors (e.g., non-specific inflammatory markers, PS score, PD-L1 expression, prognostic nutritional index), and treatment-related parameters (e.g., line of therapy, treatment strictness) (16). From this broad prognostic spectrum, we identified three core clinical variables—CRP, baseline tumor size (BTS), and PD-L1 expression—to construct a streamlined clinical model. CRP is an acute-phase protein synthesized by hepatocytes under the influence of inflammatory factors. High concentrations of CRP (>10 μg/mL) are associated with metastasis and prognosis of NSCLC and other late-stage tumors (17, 18). Regardless of the critical value of CRP or the type of variable, its baseline level is significantly associated with the prognosis of patients with advanced NSCLC receiving immunotherapy (19). Similarly, BTS directly quantifies tumor burden and functions as an independent prognostic factor, with larger size (e.g., >50 mm) correlating with significantly lower disease control rates (20, 21). PD-L1 expression as a biomarker of immunotherapy efficacy has been widely used in clinical practice. Both domestic and international guidelines recommend PD-1 inhibitors alone or in combination with chemotherapy as a first-line treatment for patients with non-small cell lung cancer (NSCLC) with negative driver gene mutations, PD-L1 high expression (TPS ≥ 50%), or PD-L1 low expression (1% ≤ TPS < 50%) (3, 22, 23). Our data confirmed their discriminative power, with responders showing significantly lower CRP and BTS and higher PD-L1 positivity. In addition, elevated LDH levels can cause lactic acid production. This may be associated with the development of cancerous tumors in the body. It is also associated with a decrease in the effectiveness of NSCLC treatment and poor prognosis (24, 25). However, the predictive value of LDH in this study was limited. Further rigorous clinical research is required to verify its potential value.

Radiomics provides a more objective, efficient, and dynamic approach than traditional manual image interpretation for assessing lesion characteristics and treatment efficacy (26, 27), with previous NSCLC immunotherapy studies demonstrating predictive value through test AUCs of 0.78-0.84 (28–31). Notably, Zhang et al. (31) confirmed through a systematic review that pre-treatment CT radiomic texture features can capture intrinsic tumor heterogeneity for efficacy prediction. They further revealed that tumors assessed as progressive at the first evaluation were characterized by abundant stroma and defective vascular structures, which impeded the infiltration of immune cells. An important methodological consideration in such studies is the strategy for lesion selection. In this regard, Wu et al. (32) demonstrated the superiority of the single-largest-lesion approach over the target-lesions method, as the latter is constrained by inter-lesional heterogeneity and clinical impracticality. Accordingly, our study adopted the single-largest-lesion approach, selecting the largest pulmonary tumor as the target lesion to ensure robustness. Building on this foundation and employing multiple machine learning algorithms, we achieved a competitive predictive performance, with AUCs of 0.926 and 0.848 in the training and testing sets, respectively.

Numerous studies have combined radiomics with diverse sets of clinical and serological markers. For instance, Miguel-Perez et al. (33) prospectively developed a combined model based on plasma PD-L1 dynamics and six radiomic features, demonstrating its potential as a biomarker for immunotherapy response. Furthermore, previous domestic and international studies (34–38) have incorporated varying clinical parameters—such as age, metastasis sites, systemic immune-inflammation index (SII), or drug types—consistently showing that combined models outperform those using clinical or radiomic features alone. In contrast to these approaches, our clinical model was deliberately constructed using a selective and distinct set of biomarkers (CRP, PD-L1, BTS). The rationale for this parsimonious feature set was rigorously validated through a formal sensitivity analysis (detailed results are provided in **Supplementary Figure 3**), which showed that incorporating seven additional common clinical covariates—namely, smoking history, ECOG PS score, neutrophil-to-lymphocyte ratio (NLR), metastasis pattern, histology, platelet-to-lymphocyte ratio (PLR), and prognostic nutritional index (PNI)—did not improve performance but slightly reduced the AUC from 0.737 to 0.712. Feature importance analysis further reaffirmed the dominant contributions of CRP, BTS, and PD-L1, indicating that other factors provided largely redundant prognostic information. This confirms that our refined clinical feature set is both optimally predictive and non-redundant. The subsequent integration of this robust clinical model with a radiomics signature (comprising one first-order and six texture features) yielded a high-performing nomogram, offering a non-invasive and efficient tool to support treatment decision-making in cancer patients.

The optimal delineation strategy for regions of interest in radiomic analysis—whether based on a single representative slice or the entire tumor volume—remains a subject of methodological discussion (39–41). While 3D segmentation theoretically provides more comprehensive spatial heterogeneity representation, 2D delineation demonstrates superior stability across different scanners and multicenter datasets, along with higher inter-observer agreement (42), our current analysis focused specifically on core tumor parenchyma to ensure feature robustness. We acknowledge that extending analysis to peritumoral regions represents a promising direction for future research to achieve more comprehensive tumor biological characterization. Combined with its closer alignment to clinical workflows through operational simplicity and standardization potential, these considerations led to our adoption of a two-dimensional annotation approach based on the largest tumor cross-section.

The nomogram demonstrated a marked performance drop from the training set (AUC = 0.953) to the initial testing set (AUC = 0.788), indicating overfitting; however, rigorous repeated cross-validation yielded a mean AUC of 0.783, closely aligning with the testing set performance and confirming a robust, generalizable discrimination ability of approximately 0.78. In contrast, calibration assessment revealed suboptimal accuracy, with visible curve deviations and a borderline Hosmer-Lemeshow test P-value (0.086) in the testing set. These discrepancies may be attributable not only to the limited validation cohort sample size but also to the inherent complexity of integrating multimodal features and potential unmodeled patient heterogeneity. Consequently, the current model is more reliable for risk stratification than for precise probability estimation. To define its clinical utility, decision curve analysis identified a probability threshold of 0.3 as optimal, at which the nomogram demonstrated substantially superior net benefit over all simple clinical benchmarks (PD-L1 expression, BTS, and CRP), confirming its significant added value for guiding immunotherapy strategies. Future efforts should focus on large-scale, external validation cohorts and algorithm refinement, incorporating *post-hoc* verification to enhance predictive calibration and secure broader clinical applicability.

An intriguing observation in this study was the notable difference in generalizability between the integrated nomogram and the standalone radiomics signature. Although both models demonstrated competent performance during training, the nomogram exhibited a more pronounced performance decline upon external validation, suggesting potential overfitting. This discrepancy appears to stem from two primary factors. First, while incorporating clinical variables aimed to enhance predictive accuracy, it may have introduced noise or cohort-specific biases, thereby promoting overfitting to non-generalizable patterns. Second, the increased model complexity from integrating multi-domain features inherently elevates overfitting risk, particularly with limited sample sizes. Moreover, radiomics features derived directly from tumor lesions may capture tumor heterogeneity more specifically and directly than indirect clinical parameters such as PD-L1 expression, BTS, and CRP, potentially accounting for their superior predictive efficacy. Although the radiomics model’s advantage in the testing set was not statistically significant (P > 0.05), its consistent performance and conceptual simplicity suggest its potential as a more robust and translatable biomarker across diverse populations. Despite demonstrating overfitting tendencies, the combined model remains clinically relevant and warrants further validation in larger, prospective cohorts.

This study has certain limitations: (1) It was a single-center, retrospective study, and PD-L1 detection using specimens from different collection sites may have affected the results, potentially leading to selection bias; (2) Manual segmentation of 2D images (central cross-sectional views of target lesions) was time-efficient and easier to perform than delineating entire tumors. However, this approach may have excluded lesions not fully captured in the maximal cross-section or those with indistinct borders that were challenging to delineate; (3) The pre-treatment contrast-enhanced CT scans were acquired using multiple scanning devices. Although image resampling and fixed gray-level discretization were applied to minimize inter-scanner heterogeneity, the variations in scanning parameters still pose a challenge to feature reproducibility. Crucially, the lack of scanner metadata in our retrospective dataset prevented us from applying the ComBat method, a powerful tool for batch effect correction, to quantify and adjust for scanner variations. Future studies should prospectively and standardly collect key information such as scanner models and scanning parameters to lay the groundwork for applying more reliable harmonization methods; (4) The study performed radiomics analysis only on primary tumors, excluding pulmonary metastases and metastases in other organs, thereby neglecting the patient survival prognosis. These factors may have introduced bias. Future efforts should include multicenter data for external validation, standardize lesion segmentation criteria, adopt semi-automated methods to enhance segmentation efficiency, and extend follow-up periods to optimize the model’s predictive performance, reliability, and generalizability.

## 5 Conclusion

In conclusion, we developed an early prediction framework for immunotherapy efficacy in advanced NSCLC using pretreatment clinical and contrast-enhanced CT radiomics features. The radiomics signature provides a robust basis for patient stratification, while the integrated nomogram shows potential for improved predictive performance, pending validation in larger cohorts.

## Data Availability

The raw data supporting the conclusions of this article will be made available by the authors, without undue reservation.
