# Supplementary material for: Early prediction of immunotherapy efficacy for advanced NSCLC based on clinical and pre-treatment contrast-enhanced CT radiomics features
Source: Front Oncol. 2025 Dec 19;15:1711402. doi: 10.3389/fonc.2025.1711402 (PMC12757218; doi:10.3389/fonc.2025.1711402)
Supplement: Supplementary file 1 [file Table1.docx]

Supplementary Material

# Model Hyperparameters and Configuration

All machine learning models were implemented using scikit-learn, XGBoost, and LightGBM libraries with fixed, pre-defined hyperparameters to ensure stability and reproducibility. The exact parameters for all algorithms are detailed in Table S1 below.

Supplementary table 1. Full Model Hyperparameters and Random Seeds

| Model | Key Hyperparameters | random_state / Reproducibility Note |
| --- | --- | --- |
| **Logistic Regression (LR)** | penalty='none', max_iter=100 | Not Applicable |
| **Support Vector Machine (SVM)** | probability=True, max_iter=100, kernel='rbf' (default) | Not Explicitly Set |
| **Random Forest (RF)** | n_estimators=50, max_depth=5, min_samples_split=4 | 0 |
| **Extra Trees (ET)** | n_estimators=20, max_depth=5, min_samples_split=2 | 0 |
| **XGBoost** | n_estimators=100, max_depth=3, min_child_weight=0.2, eval_metric='error', use_label_encoder=False | Not Explicitly Set |
| **LightGBM** | n_estimators=20, max_depth=4, min_child_weight=0.5 | Not Explicitly Set |
| **Gradient Boosting** | n_estimators=50 | 0 |
| **AdaBoost** | n_estimators=10 | 0 |

Note: For models where the random_state was not explicitly set (SVM, XGBoost, LightGBM), full computational reproducibility may require setting this parameter in future work.

# Final Model Formulae

**2.1 Radiomics Signature (Radscore):**

The formula for calculating the Radscore from the selected features is:

Radscore = 0.375 + 0.075783 * original_firstorder_Maximum + 0.009822 * original_gldm_SmallDependenceLowGrayLevelEmphasis - 0.042976 * original_glrlm_LongRunHighGrayLevelEmphasis - 0.007042 * original_glszm_GrayLevelNonUniformity - 0.033593 * original_glszm_SizeZoneNonUniformity + 0.046212 * original_glszm_SmallAreaLowGrayLevelEmphasis + 0.036860 * original_ngtdm_Coarseness

**2.2 Clinical-Radiomics Nomogram:**

The final nomogram was built using Logistic Regression to combine the clinical signature (Clinic_Sig) and the radiomics signature (Rad_Sig).

The linear predictor Z is calculated as:

Z = -2.80967098 + (3.16488672 × Clinic_Sig) + (2.82782853 × Rad_Sig)

The final predicted probability of the outcome is obtained by applying the logistic function:

P(Non-reactive = 1) = 1 / (1 + e^(-Z))

# Supplementary Figures and Tables

**Supplementary Figure 1.**

| **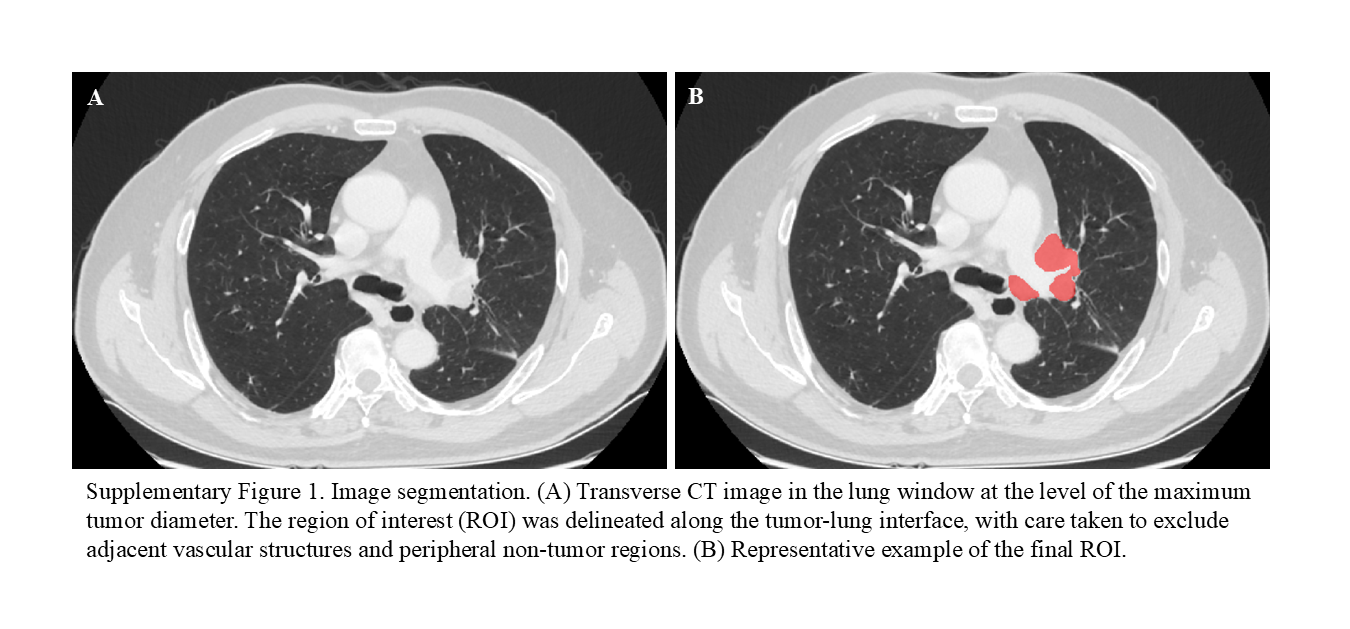** |
| --- |

**Supplementary Figure 2.**

| 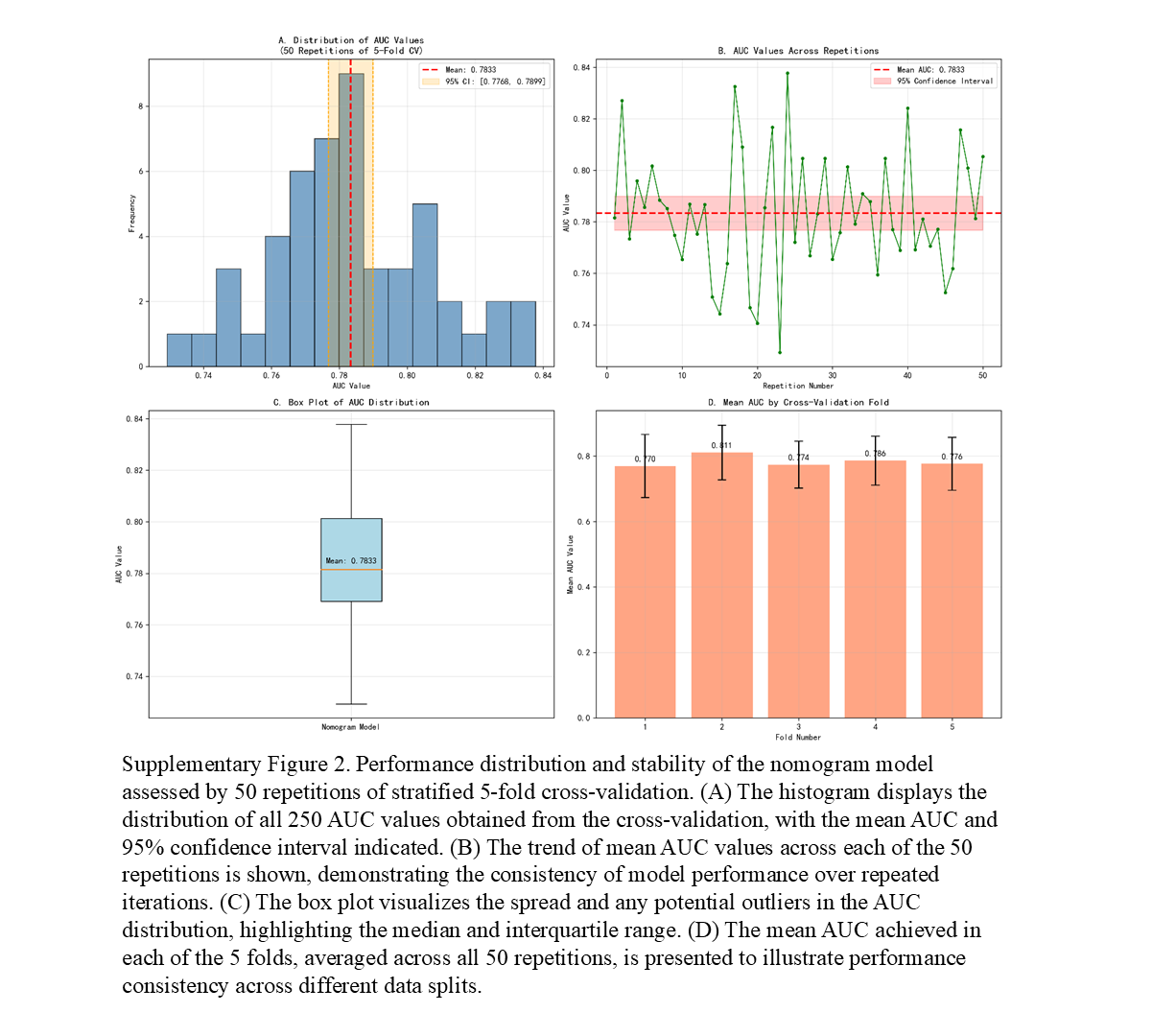 |
| --- |

**Supplementary Figure 3.**

| **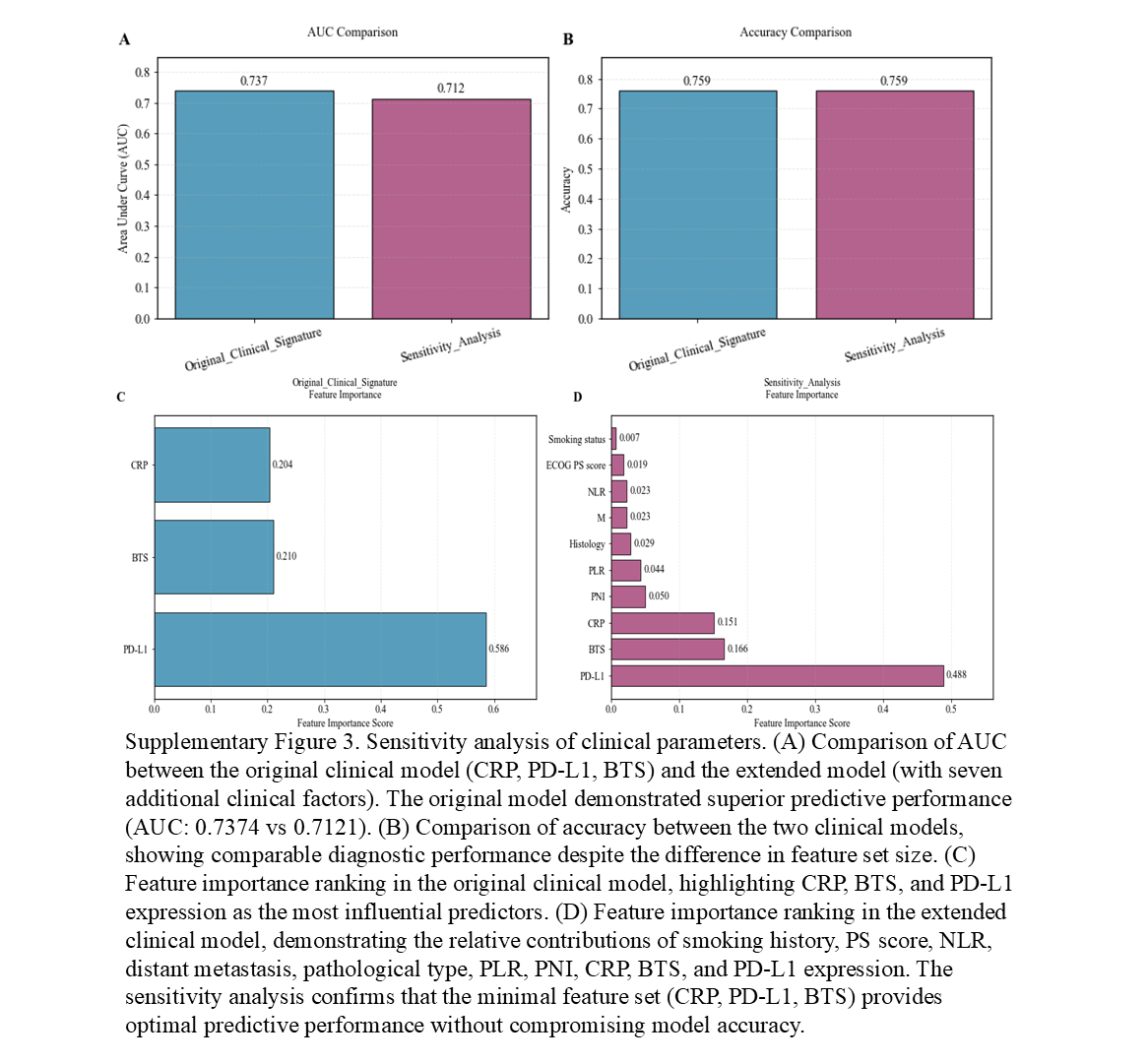** |
| --- |

**Supplementary Figure 4**

| **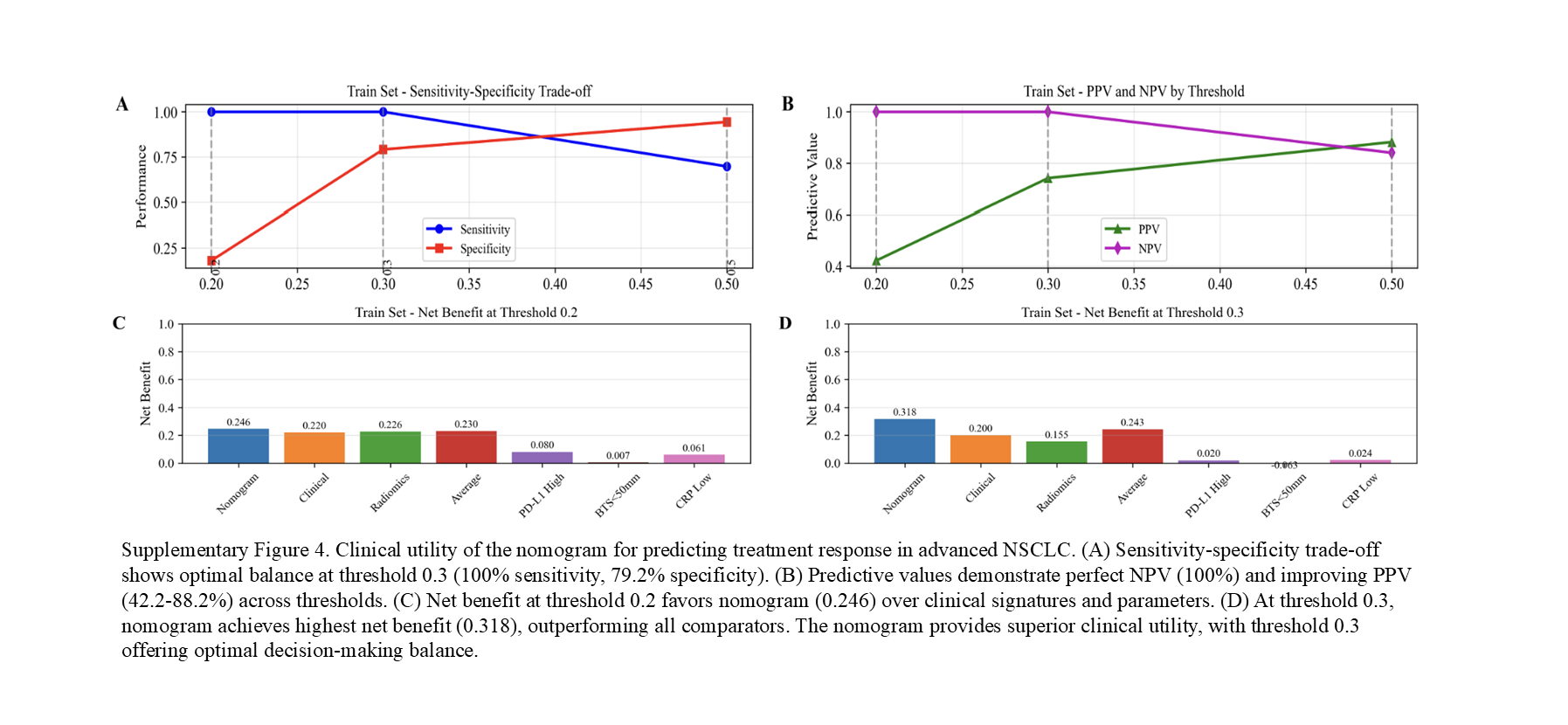** |
| --- |

**Supplementary Table 1.** **Summary of All Models Used for the Prediction of Immunotherapy Efficacy**

| Model_  name | Accuracy | AUC | 95% CI | Sensitivity | Specificity | PPV | NPV | Precision | Recall | F1 | Threshold | Task |
| --- | --- | --- | --- | --- | --- | --- | --- | --- | --- | --- | --- | --- |
| SVM | 0.975 | 0.968 | 0.906- 1.000 | 0.938 | 0.984 | 0.938 | 0.984 | 0.938 | 0.938 | 0.938 | 0.188 | label-train |
| SVM | 0.815 | 0.836 | 0.685- 0.988 | 1.000 | 0.809 | 0.500 | 1.000 | 0.500 | 1.000 | 0.667 | 0.221 | label-test |
| KNN | 0.863 | 0.883 | 0.812- 0.954 | 0.750 | 0.905 | 0.632 | 0.934 | 0.632 | 0.750 | 0.686 | 0.400 | label-train |
| KNN | 0.852 | 0.868 | 0.729- 1.000 | 0.800 | 0.905 | 0.571 | 0.950 | 0.571 | 0.800 | 0.667 | 0.400 | label-test |
| Decision  Tree | 1.000 | 1.000 | nan- nan | 1.000 | 1.000 | 1.000 | 1.000 | 1.000 | 1.000 | 1.000 | 1.000 | label-train |
| Decision  Tree | 0.741 | 0.609 | 0.355- 0.863 | 0.400 | 1.000 | 0.333 | 0.857 | 0.333 | 0.400 | 0.364 | 1.000 | label-test |
| Random  Forest | 0.988 | 0.996 | 0.986- 1.000 | 1.000 | 0.984 | 0.941 | 1.000 | 0.941 | 1.000 | 0.969 | 0.400 | label-train |
| Random  Forest | 0.852 | 0.927 | 0.831- 1.000 | 1.000 | 0.818 | 0.556 | 1.000 | 0.556 | 1.000 | 0.714 | 0.400 | label-test |
| Extra  Trees | 1.000 | 1.000 | nan- nan | 1.000 | 1.000 | 1.000 | 1.000 | 1.000 | 1.000 | 1.000 | 1.000 | label-train |
| Extra  Trees | 0.815 | 0.868 | 0.731- 1.000 | 1.000 | 0.773 | 0.500 | 1.000 | 0.500 | 1.000 | 0.667 | 0.300 | label-test |
| XG  Boost | 0.988 | 0.999 | 0.996- 1.000 | 1.000 | 0.984 | 0.941 | 1.000 | 0.941 | 1.000 | 0.969 | 0.457 | label-train |
| XG  Boost | 0.889 | 0.945 | 0.858-1.000 | 1.000 | 0.864 | 0.625 | 1.000 | 0.625 | 1.000 | 0.769 | 0.522 | label-test |
| Light  GBM | 0.863 | 0.938 | 0.889- 0.988 | 1.000 | 0.841 | 0.593 | 1.000 | 0.593 | 1.000 | 0.745 | 0.250 | label-train |
| Light  GBM | 0.815 | 0.927 | 0.822 - 1.000 | 1.000 | 0.773 | 0.500 | 1.000 | 0.500 | 1.000 | 0.667 | 0.287 | label-test |
| MLP | 0.875 | 0.904 | 0.827- 0.982 | 0.813 | 0.891 | 0.650 | 0.950 | 0.650 | 0.813 | 0.722 | 0.296 | label-train |
| MLP | 0.889 | 0.927 | 0.826- 1.000 | 1.000 | 0.905 | 0.625 | 1.000 | 0.625 | 1.000 | 0.769 | 0.326 | label-test |
| LR | 0.788 | 0.893 | 0.820- 0.965 | 0.936 | 0.750 | 0.484 | 0.979 | 0.484 | 0.938 | 0.638 | 0.151 | label-train |
| LR | 0.926 | 0.927 | 0.823- 1.000 | 1.000 | 0.952 | 0.714 | 1.000 | 0.714 | 1.000 | 0.833 | 0.525 | label-test |

**Supplementary Table 2.** **Comprehensive Evaluation of the Final Predictive Models.**

| Threshold | Sensitivity (%) | Specificity (%) | PPV (%) | NPV (%) | Accuracy (%) | Patients Identified | Threshold | Sensitivity (%) | Specificity (%) | PPV (%) | NPV (%) | Accuracy (%) |
| --- | --- | --- | --- | --- | --- | --- | --- | --- | --- | --- | --- | --- |
| 0.2 | 100.0 | 18.1 | 42.2 | 100.0 | 48.7 | 102/115 (88.7%) | 0.2 | 100.0 | 18.1 | 42.2 | 100.0 | 48.7 |
| 0.3 | 100.0 | 79.2 | 74.1 | 100.0 | 87.0 | 58/115 (50.4%) | 0.3 | 100.0 | 79.2 | 74.1 | 100.0 | 87.0 |
| 0.5 | 69.8 | 94.4 | 88.2 | 84.0 | 85.2 | 34/115 (29.6%) | 0.5 | 69.8 | 94.4 | 88.2 | 84.0 | 85.2 |
